# Assessing the unwanted: A systematic review of instruments used to assess negative effects of psychotherapy

**DOI:** 10.1002/brb3.1447

**Published:** 2019-10-24

**Authors:** Philipp Herzog, Sören Lauff, Winfried Rief, Eva‐Lotta Brakemeier

**Affiliations:** ^1^ Department of Clinical Psychology and Psychotherapy Philipps‐Universität Marburg Marburg Germany; ^2^ Marburg Center for Mind, Brain and Behavior (MCMBB) Marburg Germany

**Keywords:** assessment, instruments, negative effects, psychotherapy, side effects

## Abstract

**Objective:**

While the efficacy of psychotherapy in the treatment of mental disorders is well examined, systematic research into negative effects of psychotherapy seems comparatively rare. Therefore, this review evaluates instruments for assessing negative effects of psychotherapy in order to create a consensus framework and make recommendations for their assessment.

**Methods:**

The study selection procedure follows current best‐practice guidelines for conducting systematic reviews, with 10 included studies in three databases (PsycINFO, PubMed, and Web of Science). The nine instruments identified were each critically reviewed concerning the theoretical orientation, including the assessed domains of negative effects, psychometric properties, and diagnostic characteristics.

**Results:**

Seventeen domains of negative effects of psychotherapy were identified but inconsistently assessed by the nine instruments. Most instruments provide some initial data on their psychometric properties. Regarding diagnostic characteristics, different item‐response formats are used but often with reference to “attribution to therapy.”

**Conclusion:**

This review indicates that the existing instruments for assessing negative effects of psychotherapy cover a wide range of relevant domains without any consensus on the most important ones and their psychometric properties are usually unsatisfactory. A framework for consensus, building on the definition and conceptualization of negative effects, is synthesized, and recommendations for improving the assessment are derived.

## Graphical abstract

In contrast to the broad evidence base for psychotherapy, systematic research on assessing and reporting negative effects of psychotherapy seems rare. Through an extensive database search, nine relevant instruments measuring negative effects of psychotherapy were identified. These instruments provided to some extent psychometric data, but these efforts are still in its infancy and need further examination. A framework toward a consensus definition and conceptualization was synthesized, and future directions were given in order to improve the assessment of negative effects of psychotherapy.

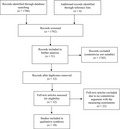

## SUMMATIONS

- Through an extensive database search, nine relevant instruments measuring negative effects of psychotherapy were identified.
- These instruments cover a wide range of relevant domains and provide to some extent psychometric data, but these efforts are still in its infancy and need further examination.
- A framework toward a consensus definition and conceptualization was synthesized, and future directions were given in order to improve the assessment of negative effects of psychotherapy.

## LIMITATIONS

- The main limitation is that this review comprises a relatively small number of eligible studies and instruments that investigate and assess negative effects.
- A further limitation is that this review focuses on psychometric properties of each instrument and therefore has not considered their clinimetric properties.

## 1 INTRODUCTION

The efficacy of psychotherapy for treating mental disorders has been well examined over several decades (Huhn et al., 2014; Schefft, Guhn, Brakemeier, Sterzer, & Köhler, 2019). In particular, the evidence base of cognitive behavioral therapy (CBT) is considered to be robust and strong (Butler, Chapman, Forman, & Beck, 2006; David, Cristea, & Hofmann, 2018; Hofmann, Asnaani, Vonk, Sawyer, & Fang, 2012). However, in comparison with research on positive effects supporting the efficacy of psychotherapy, research on negative effects is still rare. At the beginning of the 21st century, attention increased to the negative effects of psychotherapeutic interventions (Crawford et al., 2016; Scott, 2017). Psychotherapists as well as researchers highlight that negative effects are common in face‐to‐face care, for example, in group psychotherapy (Schneibel et al., 2017), as well as in Internet‐based interventions (Boettcher, Rozental, Andersson, & Carlbring, 2014). Linden et al. (2018) define negative effects1Sometimes referred to as treatment‐emergent reactions. as adverse events (AEs) related to treatment comprising side effects (SE), malpractice (MP), and unethical conduct (UC).2Formerly designated as malpractice and unethical behavior (MUB) (Ladwig et al., 2018). In contrast to MP and UC, SE are AEs caused by a correctly performed psychotherapy, that is, lege artis delivered treatment, and comprises different life domains (such as transient symptom deterioration, conflicts in interpersonal relationships, and stigmatization concerns). According to this definition, SE may be not only unexpected, but also expected and sometimes even intended effects. Accordingly, research suggests that approximately 58.7% of all patients in psychiatric hospitals, 45.2% in psychosomatic hospitals, and 93.8% in a convenience sample of former psychotherapy patients report at least one negative effect during psychotherapy (Ladwig, Rief, & Nestoriuc, 2014; Rheker, Beisel, Kräling, & Rief, 2017). This high prevalence of negative effects emphasizes the importance of evaluating negative effects not only once at the end of treatment, but also in the course of treatment and after its completion. However, recent reviews have shown that instruments assessing negative effects are heterogeneous and not systematically reported in randomized controlled trials (Jonsson, Alaie, Parling, & Arnberg, 2014), for example, in studies on persistent depressive disorder (Meister et al., 2016). In line with this result, the Consolidated Standards of Reported Trials (CONSORT) group claims that monitoring of negative effects in clinical studies on behavioral health is limited (Ioannidis et al., 2004).

Thus, despite their high prevalence, there are comparatively few systematic research studies on negative effects of psychotherapeutic interventions. Systematic research of their occurrence is hindered by a confusion of different definitions of negative effects (Parry, Crawford, & Duggan, 2016) as well as the diversity of terms and their inconsistent use (Linden, 2013), which leads to difficulties in developing adequate instruments for assessing negative effects. In this context, researchers use different terms such as “deterioration effects,” “side effects,” “negative effects,” “negative outcome,” “unwanted/undesirable effects,” “adverse events/effects,” “harm,” “mistakes,” and “treatment‐emergent reactions” synonymously, fostering confusion among researchers and psychotherapists. There are thus no instruments that are accepted worldwide as a “gold standard” and used consistently in studies. In conclusion, there is little systematic research on negative effects of psychotherapy, leading to an increasing need to improve current research methods on a sound theoretical basis.

In order to overcome current problems and the gap between their relevance and evaluation, the most recent methodological recommendations for trials of psychological interventions explicitly emphasize that the assessment of negative effects of psychotherapy should be performed using suitable methods of evaluation (Guidi et al., 2018). Thus, the main objective of this systematic review is to summarize and examine the available instruments for assessing negative effects in psychotherapy. To date, no review has focused on assessment tools, their theoretical foundation, or psychometric quality, underscoring their unique contribution to an often‐neglected research field. Moreover, the secondary objectives of the present review are (a) to create a framework of negative effects on an empirical basis, (b) to give recommendations for improving the assessment instruments, and (c) to provide an outlook on the development of future instruments, including theoretical considerations on the framework.

## 2 MATERIALS AND METHODS

### 2.1 Study selection

The entire study selection process followed the current guidelines of meta‐analyses and systematic reviews (Cuijpers, 2016). In August 2018, the first two authors conducted a computer database search of PsycINFO, PubMed, and Web of Science. The literature search was limited to the databases mentioned above, as redundancies already occurred there. The following search terms and logic were used in the database search: (Deterioration effects OR negative effects OR adverse effects OR negative treatment effects OR negative outcome OR side effects OR unwanted effects) AND (psychotherapy*) AND (instrument OR inventory OR questionnaire OR assessment OR scale OR survey). The database search was limited to “Title,” “Abstract,” and “Keywords.”

The database search with the defined parameters yielded 1,786 hits in PsycINFO, PubMed, and Web of Science (349 in PsycINFO, 233 in PubMed, and 1,204 in Web of Science). In addition, six articles were identified by reference list screening, resulting in a total of 1,792 articles. These records were carefully screened for title and abstract. Thereafter, 1,741 articles were excluded because their contents were considered unsuitable for this review. Of the remaining 51 matching hits, 19 duplicates were identified and removed for further analysis. The number of articles in the full‐text analysis was thus reduced to 32. These 32 articles were read by the first two authors, and any that did not include instruments for assessing negative effects of psychotherapy were excluded from further analysis. In total, the analysis process selected 10 studies in which nine instruments were described. All articles have been included in the qualitative synthesis of this review.

All search criteria were limited to full‐text articles published from 1986 to 2018, since the Vanderbilt Negative Indicators Scale (VNIS; Suh, Strupp, & O'Malley, 1986) was the first published structured assessment scale of negative effects of psychotherapy.

The objective of this study was to conduct a comprehensive review of current practice in the evaluation of all published qualitative and quantitative research on negative effects. Thus, studies were not excluded because of their psychometric properties, such as no data on reliability and validity or theoretical foundation. In addition, no restrictions were imposed on the place of origin of the studies, the year of publication, and the type of mental disorders presented in the sample. Nevertheless, the database search was limited to the availability of full texts in English and German.

**Figure 1:**
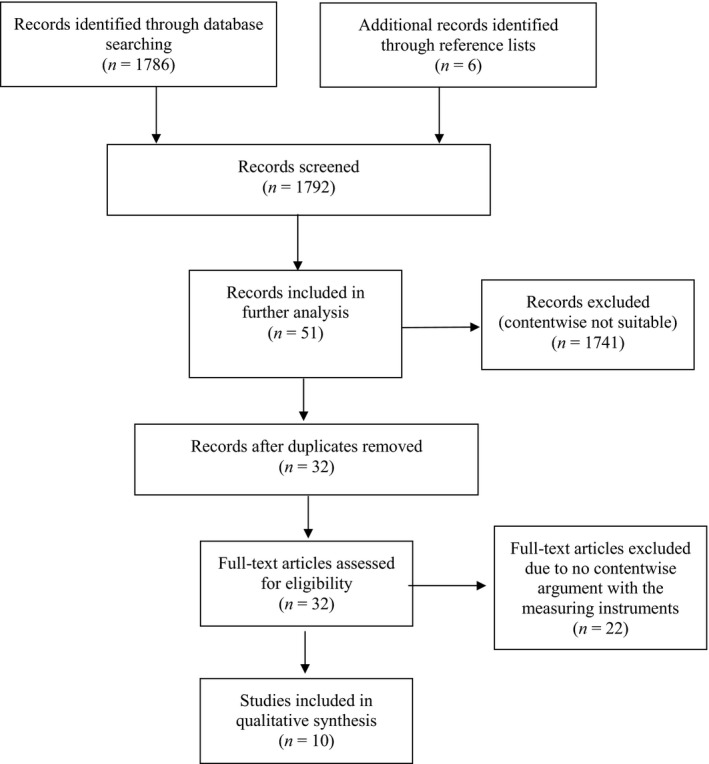
Flow diagram of included studies according to PRISMA (Moher et al., 2009)

Figure 1 displays a flowchart diagram of the selection process according to PRISMA (Preferred Reporting Items for Systematic Reviews and Meta‐Analyses; Moher, Liberati, Tetzlaff, & Altman, 2009).

### 2.2 Instrument assessment

This systematic review is based on review frameworks for the evaluation of instruments developed through the integration of standards and guidelines for psychometric evaluation, including the Journal Article Reporting Standards (APA Publications & Communications Board Working Group on Journal Article Reporting Standards, 2008) and guidelines for the evaluation of test instruments (Cicchetti, 1994). In addition, criteria for the evaluation of instruments according to Groth‐Marnat (2009) were used.

The main objective of the review framework was to identify the theoretical orientation and psychometric characteristics of the relevant instruments. Due to their theoretical orientation, the underlying theoretical construct was identified and clustered in relation to the assessed domains. To evaluate the psychometric properties of the instruments, the following components were identified as relevant within this emerging field of research: validity (content‐related, construct, predictive/criterion) and reliability (internal consistency, test–retest, inter‐rater). Diagnostic characteristics were based on number of items, item sensitivity, as well as country of origin and language versions.

All included studies were independently coded by the first two authors to allow complete extraction of relevant characteristics of each instrument and to ensure cross‐checking. The following method was used for coding: Each reviewer read the identified studies and encoded all information related to the above review framework; the extracted information from the individual studies was then discussed and systematically included in the review framework. When discrepancies and misunderstandings in coding occurred, they were resolved by discussing the information, aimed at reaching a consensus between the first two authors.

## 3 RESULTS

Table 1 presents a summary of the key characteristics of the nine identified assessment tools. The following instruments were identified: (a) Vanderbilt Negative Indicators Scale (VNIS; Suh et al., 1986); (b) Unwanted Effects–Adverse Treatment Reaction checklist (UE‐ATR; Linden, 2013); (c) Inventory for the Assessment of Negative Effects of Psychotherapy (INEP; Ladwig et al., 2014); (d) Experiences of Therapy Questionnaire (ETQ; Parker, Fletcher, Berk, & Paterson, 2013); (e) Negative Effects Questionnaire (NEQ; Rozental, Kottorp, Boettcher, Andersson, & Carlbring, 2016); (f) Unwanted Events–Adverse Treatment Reactions in the context of group psychotherapy (UE‐G; Linden, Walter, Fritz, & Muschalla, 2015); (g) Side Effects of Psychotherapy Scale (SEPS; Moritz et al., 2015); (h) Exploitation Index (EI; Epstein & Simon, 1990); and (i) Positive and Negative Effects of Psychotherapy Scale (PANEPS; Moritz et al., 2018). The VNIS, UE‐ATR, and EI are therapist‐rated instruments, while the INEP, ETQ, NEQ, SEPS, and PANEPS are patient‐rated instruments; the UE‐G is a patient‐rated instrument in the context of group psychotherapy. The EI (Epstein & Simon, 1990) was excluded for further analyses because the authors had no access to the full text in the common database and were not successful with the full‐text request. Furthermore, the UE‐ATR (Linden, 2013) was excluded for the evaluation of psychometric properties because the authors describe the checklist only as a useful, informative, and attention‐grabbing tool for recognizing negative effects, in contrast to a scale with solid psychometric properties.

**Table 1:** Summary of assessment instruments for negative effects in psychotherapy

Instrument	Author	Items	Domains	Item sensitivity	Country of origin	Language
Vanderbilt Negative Indicators Scale (VNIS)	Suh et al. (1986)	42	Unrealistic expectations Deficiencies in therapeutic commitment Inflexible use of therapeutic techniques Poor therapeutic relationship Poor match	6 points (different subscales)	USA	English
Unwanted Effects–Adverse Treatment Reaction checklist (UE‐ATR)	Linden (2013)	16	UE classes as follows: Lack of clear treatment results Prolongation of treatment Noncompliance of the patient Emergence of new symptoms Deterioration of symptoms Negative well‐being of the patient Strains in the patient–therapist relationship Very good patient–therapist relationship Strains in family relations Changes in family relations Strains in work relations Changes in the work situation Sick leave of the patient Problems in the extended social net Any change in the life circumstances of the patient Stigmatization	5 points (severity) 6 points (relation to treatment) 8 points (context of development)	Germany	German, English
Inventory for the Assessment of Negative Effects of Psychotherapy (INEP)	Ladwig et al. (2014)	21	Intrapersonal changes Therapeutic misconduct Relationship Family and friends Work Stigma	7 points (bipolar) 4 points (unipolar)	Germany	German, English
Experiences of Therapy Questionnaire (ETQ)	Parker et al. (2013)	63	Negative therapist Preoccupying therapy Beneficial therapy Idealization of therapist	5 points (strongly disagree–strongly agree)	Ireland	English
Negative Effects Questionnaire (NEQ)	Rozental et al. (2016)	32	Symptoms Quality Dependency Stigma Hopelessness Failure	2 points (yes–no) 5 points (perceived burden) 2 points (relation to treatment)	Sweden	Danish, Dutch, English, Finnish, French, German, Italian, Japanese, Norwegian, Spanish, Swedish
Unwanted Events and Adverse Treatment Reactions in the context of group psychotherapy (UE‐G)	Linden et al. (2015)	46	Group size or room Content Other group members Therapist Repercussions Global experience	5 points (extent of perceived burden)	Germany	German
Side Effects of Psychotherapy Scale (SEPS)	Moritz et al. (2015)	147	Wanted effects related to treatment Adverse treatment reactions Malpractice Unethical conduct Deterioration of illness related to treatment Treatment nonresponse related to treatment Other treatment‐emergent reaction	4 points (true–not true)	Germany	German
Exploitation Index (EI)	Epstein and Simon (1990)^a^	32	NA	4 points (never–often)	USA	English
Positive and Negative Effects of Psychotherapy Scale (PANEPS)^b^	Moritz et al. (2018)	43	Positive effects Side effects Malpractice Unethical conduct	4 points (true–not true)	Germany	German, English

a No access to full article in the common database available. In the end, the authors sent a full‐article request without success.

b PANEPS is a revised and shortened version of the SEPS.

### 3.1 Theoretical orientation

The choice of an adequate instrument for measuring negative effects of psychotherapy is often determined by its theoretical orientation with the intended use. A total of 17 domains were identified to evaluate the theoretical constructs of the individual instruments.

Table 2 presents a summary of the eight reviewed instruments for measuring common domains of negative effects. Three observations can be recorded:

1. No instruments are identical with regard to the recording of different domains;
2. No domain was covered by all instruments; and
3. Three domains were assessed by all but one instrument.

**Table 2:** Diagnostic domains of assessment instruments

	VNIS^a^	UE‐ATR^b^	INEP^c^	ETQ^d^	NEQ^e^	UE‐G^f^	SEPS^g^	PANEPS^h^
Stigma	○	●	●	●	●	○	○	●
Therapeutic misconduct	●	○	●	●	●	●	●	●
Deterioration/emergence of symptoms	●	●	●	○	●	●	●	●
Quality of therapy	●	●	○	●	●	●	●	●
Therapeutic relationship (e.g., dependency, idealization)	●	●	○	●	○	○	○	●
Expectations towards therapy	●	○	○	○	○	○	○	○
Treatment response	○	○	○	○	●	○	●	●
Intrapersonal changes	○	○	●	○	○	○	○	●
Changes and strains in life areas (e.g., work, family, relationship)	○	●	●	○	○	○	○	○
Wanted effects (e.g., benefit)	○	○	○	●	○	○	●	●
Therapy setting (e.g., room size)	○	○	○	○	○	●	○	○
Relationship to other patients	○	○	○	○	○	●	○	○
Global experience	○	○	○	○	○	●	○	○
Hopelessness	○	○	○	○	○	●	○	○
Well‐being of the patient	○	●	○	○	○	○	○	○
Noncompliance to treatment	○	●	○	○	○	○	○	○
Prolongation of the treatment	○	●	○	○	○	○	○	○

Note

● Assessed; ○ not assessed. This overview does not include the Exploitation Index (Epstein & Simon, 1990) due to no access to full‐text publication. Although the UE‐G is an instrument for measuring negative effects of group therapy, it was included in this review to identify its underlying theoretical foundation.

a Vanderbilt Negative Indicators Scale (Suh et al., 1986).

b Unwanted Effects–Adverse Treatment Reaction checklist (Linden, 2013).

c Inventory for the Assessment of Negative Effects of Psychotherapy (Ladwig et al., 2014).

d Experiences of Therapy Questionnaire (Parker et al., 2013).

e Negative Effects Questionnaire (Rozental et al., 2016).

f Unwanted Events–Adverse Treatment Reactions in the context of group psychotherapy (Linden et al., 2015).

g Side Effects of Psychotherapy Scale (Moritz et al., 2015).

h Positive and Negative Effects of Psychotherapy Scale (Moritz et al., 2018).

“Therapeutic misconduct” was assessed by all instruments except the UE‐ATR, “deterioration/emergence of symptoms” by all instruments except the ETQ, and “quality of therapy” by all except the INEP. Furthermore, “stigma” was recorded by five out of eight instruments and “therapeutic relationship (e.g., dependency, idealization)” by four out of eight, which indicates that they are relevant domains. Other different domains were assessed only by some of the instruments reviewed. “Treatment response” was assessed by NEQ, SEPS, and PANEPS, “changes and strains in life areas (e.g., work, family, relationship)” by UE‐ATR and INEP, and “wanted effects” by ETQ, SEPS, and PANEPS. Another visual analysis revealed that some domains were assessed by only one instrument. “Expectation towards therapy” was assessed only by VNIS; “intrapersonal changes” only by INEP; “therapy setting (e.g., room size),” “relationship to other patients,” “global experience,” and “hopelessness” only by UE‐G; and “Well‐being of the patient,” “noncompliance to treatment,” and “Prolongation of the treatment” only by UE‐ATR. The UE‐ATR and PANEPS have the largest overlap of negative effect indicators, with 8 out of 17 indicators.

### 3.2 Evaluation of psychometric properties

Table 3 summarizes the psychometric properties of seven instruments (without UE‐ATR). Three types of validity aspects were identified as relevant (content‐related, construct, and criterion). In addition, three types of reliability aspects were considered (internal consistency, test–retest, and inter‐rater).

**Table 3:** Evaluation of psychometric properties of instruments for measuring negative effects in psychotherapy

Instrument^a^	Studies	Total *N*	Validity			Reliability			
			Content‐related	Construct	Criterion	Internal consistency	Test–retest	Inter‐rater	Population
Vanderbilt Negative Indicators Scale (VNIS)	Strauß, Strupp, Burgmeier‐Lohse, Wille, and Storm (1992)	18	NA	NA	NA	NA	NA	0.89–0.96	Clinical
Inventory for the Assessment of Negative Effects of Psychotherapy (INEP)	Ladwig et al. (2014)	200 (195)	Literature review Consulting researchers	Factor analysis: 7‐factor solution explains 55.8% of total variance	Significant regression on satisfaction with therapy	0.86	NA	NA	Clinical
Experiences of Therapy Questionnaire (ETQ)	Parker et al. (2013) Parker, Paterson, Fletcher, McClure, and Berk (2014)	707 46	NA	Significant correlations with related constructs of the therapist satisfaction scale	NA	0.90–0.96	0.76–0.96	NA	Clinical
Negative Effects Questionnaire (NEQ)	Rozental et al. (2016)	653	Results of consensus statement Pilot study Qualitative analysis of patients' experiences	Factor analysis: 6‐factor solution explains 57.64% of total variance	NA	0.72–0.93	NA	NA	Clinical
Unwanted Events–Adverse Treatment Reactions in the context of group psychotherapy (UE‐G)	Linden et al. (2015)	71	Clinical experiences Concepts of Roback (2000) and Burlingame, MacKenzie, and Strauß (2004)	NA	NA	NA	NA	NA	Clinical
Side Effects of Psychotherapy Scale (SEPS)	Moritz et al. (2015)	173 (85)	NA	NA	NA	0.55–0.97	NA	NA	Clinical
Positive and Negative Effects of Psychotherapy Scale (PANEPS)	Moritz et al. (2018) Peth, Jelinek, Nestoriuc, & Moritz, (2018)	135	Theoretical based on the SEPS, INEP, and UE‐ATR	Factor analysis: 4‐factor solution	NA	0.72–0.92	NA	NA	Clinical

Abbreviation: NA, not available.

a The summary does not include the UE‐ATR checklist (Linden, 2013) as the authors did not aim to develop a scale with psychometric properties, or the Exploitation Index (Epstein & Simon, 1990) due to no access to the full‐article publication.

Content‐related validity was defined as the representativeness and relevance of the assessment tool for the underlying construct assessed with this tool (Groth‐Marnat, 2009). Two instruments, INEP and NEQ, showed strong content‐related validity through qualitative analysis of patients' experiences, a pilot study, a comprehensive literature review, or the advice of experts in the respective research area. In addition, PANEPS was theoretically based on INEP, UE‐ATR, and SEPS, integrating all the advantages of the individual instruments and indicating good content‐related validity. One instrument, UE‐G, also demonstrated good content‐related validity by aligning its item pool with the concepts of other researchers and their clinical experiences.

Construct validity was defined by the extent to which the instrument measures a theoretical basic construct (Groth‐Marnat, 2009). Four of the seven reviewed instruments provided relevant research data on their construct validity by means of factor analysis or correlational analysis. Three of them, INEP, NEQ, and PANEPS, provided information on the factor structure of the questionnaires, while one instrument, ETQ, showed significant correlations with related constructs.

Criterion validity was defined as comparison of the scores on the instrument with performance on another external tool (Groth‐Marnat, 2009). Only one instrument, INEP, published data on the criterion validity through regression analysis on an external criterion, in fact “satisfaction with therapy.”

Reliability of an instrument has been defined as the extent to which a score is stable, consistent, predictable, and accurate over time (Groth‐Marnat, 2009). Relevant indices of reliability are the internal consistency assessed by Cronbach's *α*, the test–retest reliability, and the inter‐rater reliability. With the exception of UE‐G, all instruments provided some reliability data, indicating a moderate to high reliability for these instruments. Alpha coefficients of .70–.79 are considered “fair,” .80–.89 as “good,” and .90 or higher as “excellent” (Cicchetti, 1994), while reliability should be at least .90 for clinical decisions and .70 for research (Groth‐Marnat, 2009). Five out of seven validation studies used the internal consistency assessed by Cronbach's *α*, which ranged from .55 to .97, indicating acceptable to excellent reliability. The test–retest reliability was only reported by ETQ, leading to values of .76–.96. The inter‐rater reliability was only given for one instrument, VNIS, resulting in values of .89–.96.

### 3.3 Diagnostic characteristics

In terms of their practical use in diagnostics, the choice of instrument for clinicians and researchers is often guided by various practical considerations such as the time of administration and user‐friendliness. The most economic self‐administered instruments in administration are the UE‐ATR and INEP, with only 16 and 21 items, respectively. With regard to item sensitivity, the results indicate the wide variety of item‐response categories used in the studies, ranging from multiple dichotomous and continuous scales used within an instrument to only one continuous item‐response category. What they all have in common is that attribution to therapy is important when recording negative effects of psychotherapy (cf. Linden et al., 2018). In line with this, the INEP, NEQ, and UE‐ATR query the relationship between negative effect and treatment. Most instruments have been developed in English‐ and German‐speaking countries and therefore are only available in English and/or German, but one instrument, the NEQ, has already been translated into several languages.

## 4 DISCUSSION

The main objective of this study was to conduct a systematic review of the current instruments for assessing the negative effects of psychotherapy by evaluating their theoretical orientation and psychometric properties, including diagnostic characteristics. This will help researchers and practitioners to select the appropriate tools for evaluating negative effects for their respective purposes, as proposed by Guidi et al. (2018). A secondary objective was to derive a bottom‐up framework of negative effects from the available data in order to refine the conception and definition of negative effects in psychotherapy and to give recommendations for improving assessment.

Overall, the results of this systematic review indicate that the available instruments for negative effects and their empirical evidence can largely be classified as insufficient, despite promising study approaches in recent years. Since 1986, only 32 studies have been published using standardized survey methods. Overall, nine instruments were identified to assess some sort of negative effects: Eight of them were studied in light of their theoretical orientation and seven of them with regard to the psychometric properties reported. The underlying theoretical constructs were clustered into 17 different domains across all instruments. Negative effects seem to be composed of several different and to some extent distinct factors. The largest coverage of the evaluated domains was achieved by the UE‐ATR. The dimension “expectations towards psychotherapy” is considered as a negative effect by only the VNIS which has been developed from a psychodynamic perspective. This dimension must therefore be interpreted under the light of its therapeutic orientation. In terms of psychometric properties, most data were available for the INEP, NEQ, and PANEPS, which all reported promising results supporting different aspects of validity and internal consistency. Since side effects are often single events rather than dimensional phenomena, side effect instruments should primarily be instruments for event monitoring. In the context of psychometric properties as empirical criterions in the assessment of side effects, therefore, the content validity seems to be most important, in the sense of assessing whether all possible side effects as events are covered by the respective scale. In addition, the PANEPS assesses both negative and positive effects of psychotherapy. With regard to practical issues, the INEP is identified as the most economic self‐report questionnaire (with 21 items) and the UE‐ATR as the most economic assessment tool for practitioners (with only 16 items).

### 4.1 Defining negative effects of psychotherapy: toward a consensus framework

This review investigated the theoretical orientation of negative effect instruments. No instrument is able to record all derived domains, and most studies lack clear definitions of negative effects. When definitions are given, they vary between studies. As there is no consensus on a model that covers all positive and negative effects of psychotherapy, assessing the diagnostic domains of all existing instruments could be useful in determining the importance of domains for building a framework. The majority of the reviewed instruments assess six core characteristics: (a) stigma, (b) therapeutic misconduct, (c) deterioration/emergence of symptoms, (d) quality of therapy, (e) therapeutic relationship (e.g., dependency and idealization), and (f) treatment response. Therapeutic misconduct is an important and very sensitive topic. Therapeutic misconduct is recorded by several instruments and can be regarded as a negative effect of an incorrectly performed therapy, whereby side effects represent negative effects of a correctly performed therapy. Of note, therapeutic misconduct can therefore never be accepted and should always lead to professional or legal consequences. Some authors argue that the wanted effects (i.e., positive effects of psychotherapy) should also be evaluated in order to minimize negative priming (Moritz et al., 2018). Negative priming may cause negative expectations about the occurrence of side effects of a particular treatment, even in psychological interventions (Bootzin & Bailey, 2005), and can therefore be associated with reported side effects—a phenomenon called the “nocebo effect,” which so far has been used mainly in psychopharmacological trials (Colloca & Miller, 2011). In recent years, more and more researchers have considered negative expectations as a key feature in mental disorders (Rief et al., 2015). By assessing the side effects of psychological interventions, these side effects might be at least partially triggered by the nocebo effect. Several authors gave initial indications on how to deal with the nocebo effect (Webster, Weinman, & Rubin, 2016), for example, by reducing expectations of symptoms or limiting symptom suggestions. In this context, the informed consent could be adapted (Cohen, 2014). It should be noted, however, that this hypothesis has not (yet) been supported by independent studies and need further empirical data in the context of psychological treatments. Patients might be “nocebo‐susceptible” to side effects, which may be interpreted as one of many patient criteria that increase the risk of side effects. Future research should pay more attention to risk factors of side effects.

In addition, researchers discuss whether positive side effects and by‐products should also be included in the framework of negative effects (Hoyer, 2016). The authors argue that the classic model of side effects in psychological interventions was derived from pharmacological models of side effects and their focus on symptom deterioration, and therefore cannot cover the complexity of the bio‐psycho‐social model of medicine and psychological interventions. The spectrum of potential negative effects in psychological interventions is greater than in pharmacological treatments, as it also includes negative events in social interactions (Szapocznik & Prado, 2007). However, research on this concept is not yet well established. For example, the improvement of quality of life was considered as one of these positive side effects, whereas other authors argue that this should always be addressed as a goal of therapy and therefore considered as a (secondary) outcome (Caspar & Jacobi, 2007). In addition, the concept of positive side effects may be misleading as most instruments also covered areas other than symptom deterioration. In light of the proposed model, positive side effects might be covered by the assessment of several domains and an indication of their valence (i.e., side effects do not necessarily have to be negative according to this definition). Therefore, future studies should consider evaluating the valence of side effects to determine their effects on treatment and outcome.

**Figure 2:**
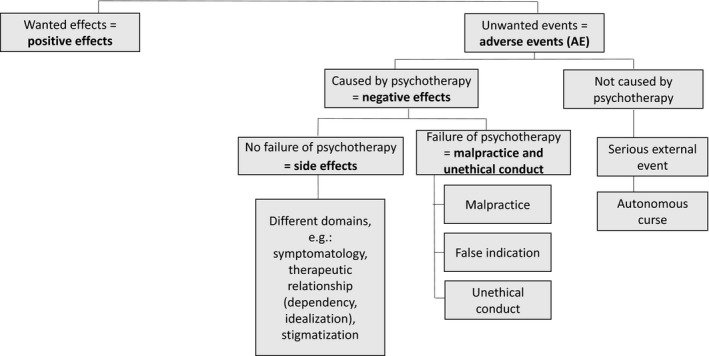
Framework for the classification of negative effects

The terms “side effects” and “negative effects” are sometimes used interchangeably in the relevant literature, leading to an inaccurate use of the technical terms. The authors therefore argue for a clear distinction between them in order to clarify the underlying constructs and to counter the confusion of different terminologies for the same construct. This should encourage the use of consistent and uniform terms in future research. It seems helpful to classify wanted effects (positive effects) and unwanted events (often referred to as adverse events) within a framework based on the previous findings of diagnostic features assessed by these instruments. In other terminology, adverse events (AEs) can be divided into treatment‐emergent reactions (AE related to treatment) and those unrelated to treatment (Linden, 2013; Moritz et al., 2018). This classification is displayed in Figure 2. On this basis, the authors try to create a consensus definition that is consistent with a recently published article by Linden et al. (2018). By integrating and synthesizing these findings within one framework, negative effects can be defined as unwanted events caused by psychotherapy. In addition, an attempt is made to distinguish between side effects and malpractice/unethical conduct. While side effects are unwanted events caused by lege artis psychotherapy (i.e., psychotherapy performed correctly), malpractice/unethical behavior can be classified as unwanted events caused by failures in psychotherapy. Side effects should include several domains, for example, not exclusively but most importantly: (a) stigma, (b) changes in symptoms (e.g., deterioration or emergence of symptoms), (c) changes and strains in life areas (e.g., work, family, relationship), and (d) therapeutic relationship (dependency and idealization). Thus, negative effects include side effects as well as malpractice and/or unethical behavior. On the other hand, wanted events caused by psychotherapy can be classified as positive effects. Furthermore, unwanted events unrelated to psychotherapy may also occur due to serious external events or the autonomic curse of the disorder. In this context, it is important to ask about the relationship between unwanted events and treatment as a crucial criterion. The various self‐report scales indicate the causal relationship to treatment by asking the patient whether the unwanted event experienced and reported was likely caused by (a) “the treatment I received” or (b) “other circumstances” (e.g., INEP and NEQ). While the UE‐ATR as a therapist rating indicates the relation to treatment on a 5‐point scale ranging from 1 = unrelated, 2 = probably unrelated, 3 = possibly related, 4 = probably related, to 5 = related. However, establishing causal relationships to the psychological interventions received is still difficult and has been a topic of discussion for decades (e.g., May, 1971).

### 4.2 Improving the assessment of negative effects

**Figure 3:**
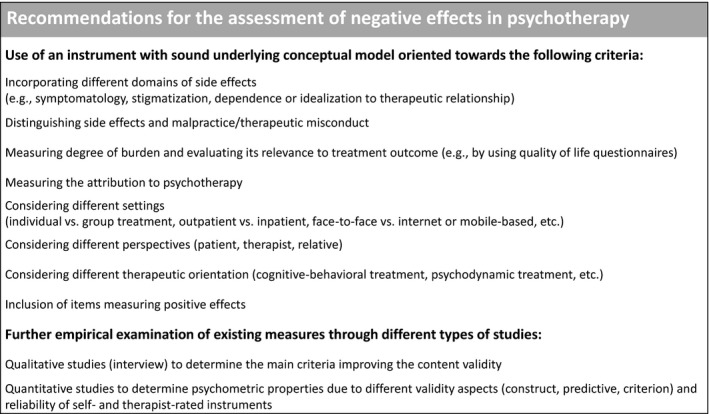
Recommendations for the assessment of negative effects

Our analysis has shown several ways to improve the assessment of negative effects. Recommendations are delineated in Figure 3. In summary, the use and development of instruments for assessing negative effects must be based on a strong theoretical background and a sound underlying conceptual model that includes a clear definition and classification of positive and negative effects, and the above‐synthesized framework might be a useful tool to comply with this. In particular, on the basis of the results of this review, the following recommendations for evaluating negative effects in psychotherapy can be derived. First, instruments need to take into account different domains of side effects (in particular stigma, symptom change, changes and strains in life areas, dependence, or idealization of the therapeutic relationship). Second, the results highlight the distinction between side effects, malpractice, and unethical conduct. The recommendation, which can be implemented by various instruments, is to use one instrument to assess side effects and another to assess malpractice and/or unethical conduct. It should be noted that the correct assessment of unethical behavior and misconduct, through both self‐report and the practitioner's report, is difficult. Thirdly, the instruments need to assess the level of burden to evaluate the relevance (and therefore impact) of side effects and also assess the attribution to psychotherapy; therefore, future studies should consider evaluating the relevance of side effects to determine their effects on treatment and outcome. With the exception of INEP, no other reviewed instrument reported data on important criterion validity aspects. Criterion validity can be measured by scales that assess treatment outcome in terms of specific and general symptom reduction or the patients' quality of life, for example. Various instruments are available to assess symptom reduction and the quality of life (e.g., SF‐36: Zwingmann, Metzger, and Jäckel (1998), WHOQOL‐BREF: The WHOQOL Group (1998)), which should be used in future studies. Fourthly, when assessing side effects, researchers must consider the setting (individual and/or group treatment, outpatient or inpatient, face‐to‐face, Internet or mobile‐based, etc.), the perspective (patient, therapist, relative), and the therapeutic orientation (cognitive behavioral treatment, psychodynamic treatment, etc.). Depending on the therapeutic orientation, it can vary whether events are regarded as side effects or part of an effective therapy. For example, CBT therapists may describe a deterioration of symptoms or dependence on the therapist as a side effect rather than a psychoanalyst, who may regard it as a component of the effective therapy. At best, instruments should be developed by scientists and practitioners of different orientations, which has not been done so far with the instruments of this review. Most instruments were developed and validated in the context of CBT, while only the VNIS was developed with a psychodynamic focus. Therefore, some important constructs of psychotherapeutic effects in general might not have been covered by the strong focus on CBT, such as the therapeutic relationship. Future research should therefore conduct studies with a broader therapeutical background by incorporating also mental health services with a focus on, for example, psychoanalytical and psychodynamic therapy as well as so‐called “Third Wave” therapies. While it is important for the recording of negative effects that appropriate instruments are used in prospective (descriptive) studies, it also seems to be very useful to develop strategies on how negative effects can be prevented. For example, it could be investigated whether the involvement of significant others in treatment at an early stage and homework to strengthen autonomy can counteract dependence on the therapist. In addition, psychotherapists should be trained in the detection, monitoring, and minimization of possible side effects. Fifth, the positive effects of psychotherapy—in contrast to the concept of positive side effects—should also be taken into account when assessing side effects in order to minimize the potential of the aforementioned nocebo effects.

To realize this recommendation, a suitable assessment tool for measuring side effects of psychotherapy may be embedded in the broader context of the so‐called Routine Outcome Monitoring (ROM). ROM has been shown to be effective at reducing treatment failure and likewise enhancing the positive effects of psychotherapy (Lambert & Harmon, 2018; Lambert, Whipple, & Kleinstäuber, 2018). ROM therefore yields some considerable merit for the implementation of evidence‐based practice in routine care, and the assessment of side effects may broaden and enrich current ROM strategies. Moreover, further empirical examination of existing measures through different types of studies is needed, that is, qualitative studies (interview) to determine the main criteria improving the content validity and quantitative studies to determine psychometric and clinimetric properties due to different validity aspects (construct, predictive, and criterion) and reliability of both self‐ and therapist/observer‐rated instruments.

### 4.3 Limitations of the review

The first limitation of this systematic review concerns the relatively small number of eligible studies and instruments that investigate and assess negative effects. Secondly, this review included all available assessment tools of negative effects (e.g., UE‐G is an instrument that only measures the negative effects of group psychotherapy). Since there has been no consensus on negative effects so far, the heterogeneity of the examined instruments may be considered as one limitation of this review. However, the authors adhere to this approach to do an exhaustive search and examine all relevant underlying theoretical foundations in order to extract the diagnostic features and synthesize a comprehensive model. Thirdly, this review did not examine the clinimetric properties of each instrument (Bech, 2016), which might be especially important in terms of research on psychological interventions (Fava, Rafanelli, & Tomba, 2012). Fourthly, in psychotherapy outcome research, the reliable change index (RCI) is used extensively for defining deterioration using standardized rating scales (Jacobson, Follette, & Revenstorf, 1984). The RCI has not been considered in this review because the scope of this review was to study assessment tools of negative effects during the course of therapy or after completion. In general, negative effects were considered more as a process variable than an outcome variable. Within this framework, deterioration as one potential side effect might not be seen as an outcome, more as a transient and short‐term effect that may occur during the therapy process. Finally, the lack of variability of the patients participating in these studies could be another limiting factor narrowing to some extent the use of such instruments across highly heterogeneous mental health issues. For example, patients with more severe psychiatric disorders (such as personality disorder or schizophrenia) may experience more serious side effects than patients with less severe disorders (such as mild depression and no comorbidities). In this context, first studies suggest that inpatients who are usually more severely ill report more side effects than outpatients (see Brakemeier et al., 2018; Rheker et al., 2017). Future studies should therefore specifically include severely ill patient groups in order to identify specific negative effects and compare them between different patient groups.

### 4.4 Future research directions

There are several future directions for improving the assessment of negative effects of psychotherapy. First, existing instruments need to be evaluated with regard to their psychometric properties (see Figure 3). Of note, psychometric research was mainly developed outside the clinical field and although psychometrics has been used successfully in clinical psychology research and has led to some advances in evaluation, it has guided research to rely strongly on its advantages and to neglect its disadvantages. Thus, when developing new assessment tools in future research the clinimetric properties should be considered (Bech, 2016; Fava et al., 2012; Feinstein, 1987): those features of an instrument that identify clinically relevant changes in mental health over time (discrimination properties such as responsiveness/sensitivity; Fava, Tomba, & Bech, 2017) and predict long‐term incremental validity within the clinical decision‐making process (Fava et al., 2012). In the case of negative effects, besides the evaluation of psychometric properties, they should be linked to treatment outcome in order to determine the impact (relevance) of treatment on the individual patient's life. For example, within process–outcome research, future studies could link the occurrence of negative effects to treatment outcome, for example, by using the RCI (Jacobson & Truax, 1991). Initial attempts have been made to address the relevance of negative effects on treatment in an inpatient cognitive behavioral analysis system of psychotherapy (CBASP) sample (Brakemeier et al., 2018). In line with this, the current methodological recommendations for trials of psychological interventions support the usefulness of clinimetrics (Guidi et al., 2018). Second, there is considerable need to develop new instruments for assessing negative effects in specific populations (e.g., children and adolescents) and for different settings (e.g., short forms and specific items for group therapy and inpatient use). Third, most instruments are self‐rated; thus, validated clinician‐rated instruments would be valuable to provide therapists with a standardized tool to monitor negative effects during treatment. A promising approach is the UE‐ATR, which should be validated in future studies. Fourth, longitudinal research designs could provide insights into the predictive validity of instruments (including clinimetric, discriminant, and incremental validity) as well as to improve our understanding of the influence of negative effects on treatment outcome (i.e., response, remission, relapse, and dropout) in order to determine the relevance of the negative effects (cf. Brakemeier et al., 2018). The prevalence of negative effects seems to vary widely from study to study, depending on the selection of instrument (Ladwig et al., 2014; Moritz et al., 2015, 2018; Rheker et al., 2017); therefore, an instrument that is recognized worldwide as the “gold standard” is desirable for use in most studies in order to make study results comparable. In addition, current methodological guidelines for trials plead for the assessment of negative effects of psychotherapy using suitable evaluation methods (Guidi et al., 2018). In order to monitor and counteract negative effects, in particular side effects, further studies need to develop a process scale that assesses negative effects during therapy and with a clear time frame. This would strengthen the clinimetric properties and thus clinical usefulness of an instrument in clinical practice. Further, such a process scale that regularly assesses side effects of psychological interventions may be a useful extension for ROM (Lambert & Harmon, 2018). The aim should always be to carry out effective psychotherapies with as few side effects as possible.

## CONFLICT OF INTEREST

The authors declared no conflicts of interest.

## AUTHORS' CONTRIBUTION

All authors have been significantly involved in the research and/or article preparation. All authors have approved the final article.

## Data Availability

Data sharing is not applicable to this article as no new data were created or analyzed in this study.
